# Zinc Finger and BTB Domain-Containing 20: A Newly Emerging Player in Pathogenesis and Development of Human Cancers

**DOI:** 10.3390/biom14020192

**Published:** 2024-02-04

**Authors:** Jiangyuan Liu, Han Zhang

**Affiliations:** Institute of Medical Biology, Chinese Academy of Medical Sciences and Peking Union Medical College, Kunming 650118, China; liujiangyuan@student.pumc.edu.cn

**Keywords:** ZBTB20, transcription factor, human, cancer, hematological malignancy

## Abstract

Zinc finger and BTB domain-containing 20 (ZBTB20), which was initially identified in human dendritic cells, belongs to a family of transcription factors (TFs) with an N-terminal BTB domain and one or more C-terminal DNA-binding zinc finger domains. Under physiological conditions, ZBTB20 acts as a transcriptional repressor in cellular development and differentiation, metabolism, and innate immunity. Interestingly, multiple lines of evidence from mice and human systems have revealed the importance of ZBTB20 in the pathogenesis and development of cancers. ZBTB20 is not only a hotspot of genetic variation or fusion in many types of human cancers, but also a key TF or intermediator involving in the dysregulation of cancer cells. Given the diverse functions of ZBTB20 in both health and disease, we herein summarize the structure and physiological roles of ZBTB20, with an emphasis on the latest findings on tumorigenesis and cancer progression.

## 1. Introduction

**Figure 1:**
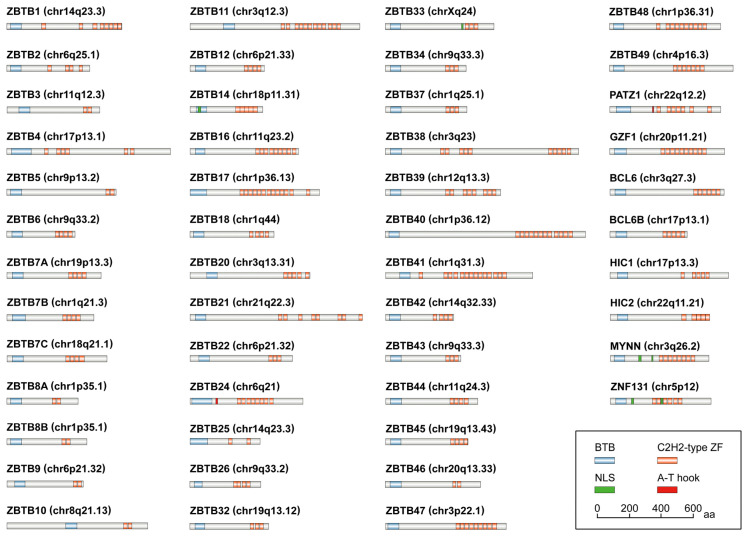
Schematic representation of ZBTB family members and their encoded domains. The sequences of ZBTB proteins and their functional information are obtained from the UniProk Knowledgebase (UniProtKB) [10] and National Center for Biotechnology Information (NCBI). aa, amino acids; BCL6, BCL6 transcription repressor (also known as ZBTB27); BCL6B, also known as ZBTB28; BTB, bric-a-brac/tramtrack/broad complex; C2H2-type ZF, C-terminal Cys2His2/Krüppel-type zinc finger; chr, chromosome; GZF1, GDNF-inducible zinc finger protein 1 (also known as ZBTB23); HIC1, HIC ZBTB transcriptional repressor 1 (also known as ZBTB29); HIC2, also known as ZBTB30; MYNN, myoneurin (also known as ZBTB31); NLS, nuclear localization signal; PATZ1, POZ/BTB and AT hook-containing zinc finger 1 (also known as ZBTB19); ZNF131, zinc finger protein 131 (also known as ZBTB35).

Zinc finger and BTB domain-containing (ZBTB) proteins are an evolutionarily conserved family of transcription factors (TF), characterized by the presence of an N-terminal BTB domain and one or more C-terminal Cys2His2 (C2H2)/Krüppel-type zinc finger (ZF) domains [1]. So far, at least 49 ZBTB proteins have been identified in the human genome (Figure 1), exhibiting diverse functions in both physiological and pathological conditions [1]. Of these members, ZBTB20 was initially identified as a dendritic cell (DC)-derived ZBTB protein (also named DPZF, HOF, or ZNF288) [2], acting mainly as a transcription repressor involved in many biological processes, including cellular differentiation, developmental regulation, innate immunity, and metabolism [3,4,5,6,7,8,9]. ZBTB20 has also been increasingly identified as a key regulator in the pathogenesis and development of human cancers. However, ZBTB20 has seldom been specifically reviewed in terms of its function in tumorigenesis. As such, we summarize the structure and physiological function of ZBTB20, with a particular focus on its roles in human cancers and potential therapeutic implications.

## 2. Functional Domains of ZBTB Proteins

### 2.1. The BTB Domain

The BTB domain, also known as the POZ (poxvirus and zinc finger) domain, is a conserved protein–protein interaction motif widely distributed in the proteins of higher eukaryotes [11]. It has approximately 100–120 amino acids (aa) within the N terminus of ZBTB protein (Figure 1). Despite being structurally well conserved [12], the BTB domain participates in a variety of cellular processes. It mediates not only the formation of homodimers, heterodimers, or multimers [12,13,14], but also the recruitment of transcriptional regulators [15,16], hence allowing for diverse functions, ranging from transcriptional repression [17,18,19] and cytoskeleton dynamics [20] to protein ubiquitination and degradation [21,22,23,24]. BTB domains are also capable of regulating gene expression through chromatin remodeling [25]. Given its essential roles, the BTB domain has become a promising target for some diseases. Numerous studies have demonstrated the successful pharmacological inhibition of the BTB domain by small-molecule and peptide-mimicking compounds [26,27,28,29,30,31], although its therapeutic use needs further evaluation.

### 2.2. The C2H2-Type ZF Domain

The C2H2-type ZF domains are one of the most common DNA-binding motifs found in the TFs of higher eukaryotes [32]. The classical C2H2 domain has 25–30 aa with a left-handed ββα structure, which consists of a β-hairpin followed by an α-helix. Although C2H2 domains contain highly conserved cysteine and histidine pairs, the other aa residues are variable [32], and the number of C2H2-type ZF domains in each ZBTB protein varies too (Figure 1). These variations enable ZBTB proteins to have a unique DNA-binding capability in a sequence-specific manner [33,34]. Moreover, C2H2-type ZFs also mediate protein–protein interactions. For example, the ZFs of ZBTB16 (also known as PLZF) mediates its interaction with GATA-binding protein 2 (GATA2) [35]. The association between ZBTB16 and the histone acetyltransferase E1A-binding protein p300 (EP300) also depends on the acetylation of lysines in ZFs of ZBTB16 [36].

### 2.3. Other Known Domains

In addition to BTB and ZF domains, further sequence analyses have revealed other motifs or domains. So far, ZBTB14, ZBTB33, myoneurin (MYNN, also known as ZBTB31), and zinc finger protein 131 (ZNF131, also known as ZBTB35) have been identified to have one or two nuclear localization signals (NLS) (Figure 1), which are short peptides that act as a signal fragment to mediate the transport of proteins from the cytoplasm into the nucleus [37]. In addition, ZBTB24 and POZ/BTB and AT hook-containing zinc finger 1 (PATZ1, also known as ZBTB19) contain an A-T hook (Figure 1), a DNA-binding domain that interacts with the minor groove of AT-rich regions, thus facilitating the binding of other TFs [38,39]. Specifically, the A-T hook domain of ZBTB24 is essential for heterochromatin localization [40], whereas the region containing the A-T hook of PATZ1 is sufficient to interact with ring finger protein 4 (RNF4), a transcriptional repressor [41].

### 2.4. The Linker Region

In nearly all ZBTB proteins, the BTB and ZF domains are connected by a long linker region, which is predicted to be unstructured in most cases and confers flexible DNA-binding properties [12]. However, very limited studies have been carried out in this field. Based on the existing research, the linker region appears to act as a key mediator with specific effects on the regulation of DNA-binding capacity and protein stability [1].

### 2.5. ZBTB Family Members

In humans, the ZBTB family comprises a large group of TFs, many of which serve as master regulators of developmental events. Among them, BCL6 transcription repressor (BCL6), also known as ZBTB27, is a hallmark member controlling germinal center (GC) formation and T follicular helper cell differentiation [42,43,44,45,46]. By contrast, ZBTB16 directs the function and development of natural killer T (NKT) cells [47,48]; ZBTB20 promotes plasma cell differentiation but restricts T-cell memory differentiation [7,9]; ZBTB32 is essential for the proliferation of NK cells and limits the duration of antibody recall responses [49,50]. Due to their pivotal roles in hematopoiesis and immune responses, it is not surprising that abnormal expression or dysfunction of ZBTB proteins leads to hematological malignancies. Indeed, the first characterized mammalian ZBTB protein was from the study of chromosomal translocations in human acute promyelocytic leukemia (APL). Analysis of the t (11;17) translocation from a case with APL led to the discovery of *ZBTB16*, which is fused to the retinoic acid receptor alpha (*RARA*), resulting in the worst prognosis in APL [51]. Another example is the deregulated BCL6 expression which not only causes B-cell lymphoma development [52] but also contributes to leukemia initiation [53,54]. Consequently, BCL6 has become an attractive therapeutic target for B-cell lymphomas and leukemias [54]. In addition to hematological malignancies, ZBTB family proteins are also involved in skeletal abnormalities [55,56], infertility [57,58,59], and neurological disorders [60,61,62].

Of ZBTB family members, *ZBTB20* encodes a 741-residue protein with an N-terminal BTB domain and 5 C2H2-type ZF domains at the C terminus [63]. Notably, *ZBTB20* is localized on the chromosome 3 where *BCL6* is located and shares high homology to *BCL6* with the identity of 56% in the BTB domain and 40% in the C2H2-type ZF domain; particularly, ZBTB20 is widely expressed in hematopoietic tissues [2]. These similarities imply that ZBTB20 may be highly close to BCL6 with a certain role in hematopoiesis, immune responses, and oncogenesis. 

## 3. Physiological Roles of ZBTB20

### 3.1. Lymphoid Development and Differentiation

**Figure 2:**
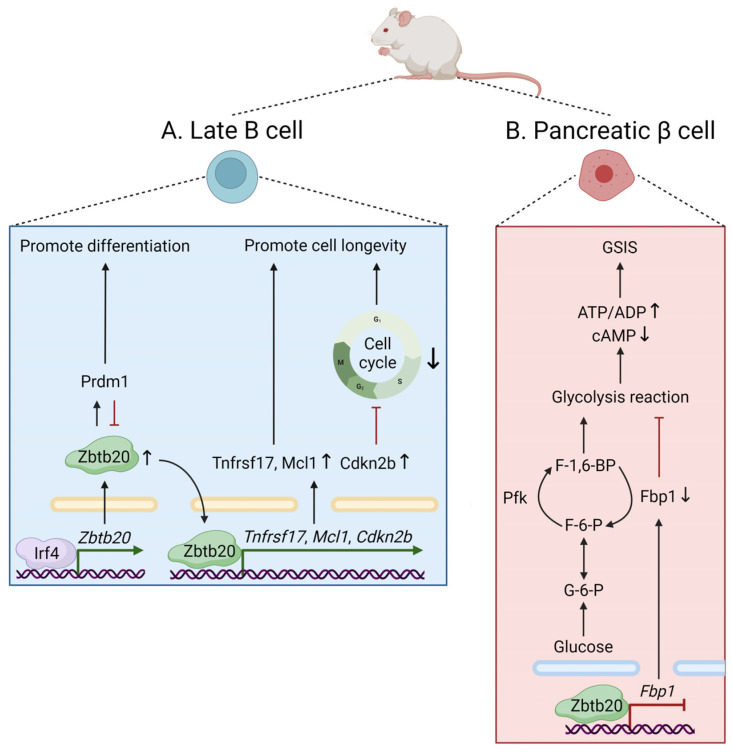
The physiological roles of Zbtb20 in mice. (**A**) Zbtb20 facilitates plasma cell differentiation via an Irf4/Zbtb20/Prdm1 axis, in which Zbtb20 induction does not require Prdm1 but depends on Irf4 by directly binding to the *Zbtb20* promoter. Zbtb20 also induces cell survival and blocks cell cycle progression by regulating the *Tnfrsf17*, *Mcl1*, and *Cdkn2b* genes, thus promoting plasma cell longevity. (**B**) In murine pancreatic β cells, Zbtb20 regulates glucose sensing and insulin secretion by repressing *Fbp1*, which encodes a gluconeogenic enzyme that regulates glucose metabolism and insulin secretion in β cells. ADP, adenosine diphosphate; ATP, adenosine triphosphate; cAMP, cyclic adenosine monophosphate; Cdkn2b, cyclin-dependent kinase inhibitor 2B; F-1,6-BP, fructose-1,6-bisphosphate; F-6-P, fructose-6-phosphate; *Fbp1*, fructose-1,6-bisphosphatase 1; G-6-P, glucose-6-phosphate; GSIS, glucose-stimulated insulin secretion; Irf4, interferon regulatory factor 4; Mcl1, myeloid cell leukemia sequence 1; Pfk, phosphofructokinase. Prdm1, PR domain-containing 1, with ZNF domain; Tnfrsf17, tumor necrosis factor receptor superfamily, member 17.

ZBTB20 was originally identified in human DCs and widely expressed in hematopoietic tissues including the spleen, lymph node, thymus, peripheral blood cells, and fetal liver [2]. In mice, Zbtb20 is highly expressed in B1 and GC B cells and reaches its highest level in mature plasma cells in the bone marrow (BM) [7]. Of note, ectopic expression of Zbtb20 in primary B cells facilitates plasma cell differentiation, whereas in plasma cells, Zbtb20 promotes cell longevity by inducing cell survival and cell cycle arrest (Figure 2A) [7]. These roles are opposite to those played by Bcl6, reflecting a possible competition between Zbtb20 and Bcl6 for binding to common gene targets. Moreover, Zbtb20 is also required for long-term antibody production and plasma cell persistence, specifically after alum-adjuvanted immunization [8], revealing Zbtb20 as a potential molecular determinant to drive durable antibody response.

In addition to B-cell development, *Zbtb20*-deficient CD8^+^ T cells exhibit upregulated mitochondrial metabolism and glycolysis, which skew CD8^+^ T cells toward the memory differentiation and enhanced secondary responses [9]. Intriguingly, memory CD8^+^ T cells lacking Zbtb20 confer superior protection against tumors compared to wild-type (WT) memory cells [9], suggesting that deletion or inhibition of Zbtb20 in CD8^+^ T cells may provide a promising strategy for anti-tumor immunotherapy. Very recently, the same research group extended the analysis to the transcriptional and epigenetic landscapes of *Zbtb20*-deficient CD8^+^ T cells at both the effector and memory phases [64]. They found that memory CD8^+^ T cells lacking Zbtb20 are enriched for the expression of numerous activator protein-1 (AP-1) components and exhibit a striking epigenetic signature associated with the attenuation of T cell activation. The above findings highlight the unique roles of Zbtb20 in T-cell lineage distinct from those in B cells.

Apart from lymphoid differentiation, ZBTB20 is also involved in the functional roles of regulatory B/T (Breg/Treg) cells. In mice, *Zbtb20* is upregulated in Breg cells from all organs, suggesting that *Zbtbt20* may serve as a potential marker gene for Breg cells [65]. By contrast, Zbtb20 expression defines the function of a subset of Treg cells involved in intestinal integrity [66]. Specifically, nearly half of Zbtb20-expressing (Zbtb20^+^) T cells express forkhead box protein P3 (FoxP3), a lineage-defining transcription factor for Treg cells. These Zbtb20^+^ Treg cells are enriched in the intestine and constitutively transcribe interleukin 10 (*IL10*), a key cytokine for modulating intestinal homeostasis. As such, Zbtb20 conditional knockout mice display severe intestinal inflammation and damage in response to induction of acute colitis, whereas adoptive transfer of Zbtb20^+^ Treg cells, but not non-Zbtb20 Tregs, rescues the mice from colitis. However, apart from the intestine, Zbtb20^+^ Treg cells with a similar ability to produce IL10 are also detected in the thymus and spleen, indicating that Zbtb20^+^ Tregs are a committed population rather than an induced effector.

### 3.2. Cellular Metabolism

In addition to T-cell immunometabolism mediated by Zbtb20, as mentioned [9], multiple lines of evidence have revealed the function of ZBTB20 in the regulation of cellular metabolism. In *Zbtb20*-null mice, transcriptional profiling of liver tissue reveals significant alterations in the expression of genes involved in glucose metabolism [3]. Further analysis revealed that Zbtb20 is abundantly expressed in pancreatic β cells and plays a key role in regulating glucose sensing and insulin secretion via transcriptional repression of fructose-1,6-bisphosphatase 1 (*Fbp1*), a regulator of glucose metabolism and insulin secretion in β cells [5] (Figure 2B). In hepatocytes, Zbtb20 also regulates plasma triglyceride metabolism by repressing the transcriptional activity of lipoprotein lipase, highlighting a critical role of ZBTB20 for hepatic lipogenesis in mice [67]. Such metabolic disorders mediated by ZBTB20 become more evident in patients with Primrose syndrome, an autosomal dominant disease caused by heterozygous missense variants in *ZBTB20*, manifested by multisystem failures including disturbed lipid and glucose metabolism as well as mitochondrial dysfunction [68,69].

### 3.3. Neurodevelopment

In central nervous system (CNS), Zbtb20 was initially characterized in hippocampal neurons, cerebellar granule neurons, and macroglia, and two Zbtb20 isoforms, designated *Zbtb20(S)* and *Zbtb20(L)*, were identified [63]. Ectopic expression of Zbtb20(S) and/or Zbtb20(L) in non-hippocampal immature pyramidal neurons induces hippocampus (Hi)-like cortical neurogenesis in the mouse brain, but similar phenotypes are observed in Zbtb20(S), Zbtb20(L), and Zbtb20(S/L) transgenic mice, suggesting an overlap in function of these two isoforms [70]. Furthermore, Zbtb20 has a dynamic expression pattern in the germinative zones of the developing neocortex and serves as a regulator of the timed sequential production of distinct neuronal fates during cortical neurogenesis [71].

Specifically, Zbtb20 is essential for the specification of hippocampal Cornu Ammonis 1 (CA1) field identity and the postnatal survival of hippocampal neurons [62]. The mice with specific deletion of *Zbtb20* in mature CA1 pyramidal neurons exhibit no obvious morphological abnormalities but display impaired Hi-dependent memory, demonstrating that Zbtb20 is critical for both the specification of CA1 field identity in the developing Hi and Hi-dependent long-term memory formation in mature CA1 cells [72]. Subsequent studies further uncovered that Zbtb20 is highly expressed in all the mature endocrine cell types of anterior pituitary, and its deficiency impairs anterior pituitary development. *Zbtb20*-null mice also display severe defects in lactotrope specification and lineage expansion, pinpointing that Zbtb20 is a crucial determinant of lactotrope specification [73]. Recently, the same group dissected the roles of Zbtb20 in mature lactotropes. Interestingly, Zbtb20 is dispensable for the homeostasis of lactotrope relative density in adult pituitary or the expansion elicited by pregnancy and lactation in females but is required to maintain prolactin expression and lactotrope function, highlighting a critical role of Zbtb20 in mature lactotropes in adult mice [74].

### 3.4. Immune Response and Inflammation

Being a master regulator of lymphoid development and differentiation, ZBTB20 plays a key role in immune response and inflammation. For instance, Zbtb20 promotes full activation of Toll-like receptor (TLR) signaling, a critical pathway in innate response against invading pathogens; mechanistically, Zbtb20 enhances NF-κB activation by specifically repressing the transcription of *Nfkbia* (*IκBα*), the canonical suppressor of NF-κB signaling, thus promoting TLR-triggered production of proinflammatory cytokines and type I interferon (IFN) in macrophages [6]. Consistent with this study, ZBTB20 affects the outcome of chlamydia and salmonella infections through the suppression of multiple target genes including *NFKBIA* [75]. ZBTB20 has also been revealed to regulate cardiac allograft rejection via macrophage polarization and NF-κB-mediated inflammation [76]. Other than ZBTB20, circular RNA (circRNA) *circZbtb20* has been newly identified to promote the homeostasis and function of group 3 innate lymphoid cells (ILC3s), a group of innate effectors involved in host defense against pathogens at the early stage [77]. All these findings pinpoint that ZBTB20 is a pleotropic regulator of immune response and inflammation. The above-mentioned physiological roles of ZBTB20 are summarized in Table 1.

## 4. ZBTB20 in Cancers

Despite the involvement of ZBTB20 in various cellular processes under healthy conditions, understanding of the roles of ZBTB20 in malignancies is still in its infancy. Over the past decades, ZBTB20 has been well researched in liver cancer even though most elegant investigations were limited to mice. In addition, many *ZBTB20* variants have been identified in some cancers, but the pathogenic mechanisms are poorly understood. In this case, we summarize the known mechanisms and potential functions of ZBTB20 in different cancers (Table 2).

### 4.1. Hepatocellular Carcinoma

Historically, alpha-fetoprotein (AFP) has been well recognized as a serum biomarker for hepatocellular carcinoma (HCC) and some other cancers. Interestingly, Zbtb20 is a potent transcriptional repressor of the *Afp* gene in mouse liver. In liver-specific *Zbtb20* knockout (Zbtb20^KO^) mice using the Cre/loxP approach, efficient deletion of the *Zbtb20* gene results in a dramatic increase in *Afp* transcription in the entire liver throughout adult life [78]. Further analysis revealed that the *Afp* gene harbors a cognate Zbtb20-binding site at −104/−86, which mediates sequence-specific binding and subsequent repression by Zbtb20 in mouse liver [79]. Of note, specific ablation of Zbtb20 in postnatal hepatocytes does not compromise liver development [78], but the mice specifically lacking Zbtb20 in hepatocytes exhibited a remarkable defect in liver regeneration after partial hepatectomy due to impaired hepatocyte proliferation and delayed cyclin D1 induction [80]. Mechanistically, the defect in liver regeneration is mainly attributed to the Zbtb20-epithelial growth factor receptor (Egfr) signaling, in which Zbtb20 deficiency substantially decreases hepatic expression of Egfr, a critical regulator of efficient liver regeneration [81], highlighting a potential role of the Zbtb20-Egfr axis in hepatocellular carcinogenesis [80]. This finding is supported by a previous work using a conditional transposon-based insertional mutagenesis screen which identified *Zbtb20* and *Egfr* as 2 of 19 highly significant candidate loci implicated in causing HCC [82]. Nonetheless, genetic deletion of hepatic *Afp* does not affect liver regeneration in liver-specific Zbtb20^KO^ mice, suggesting that Zbtb20 regulates liver regeneration in an AFP-independent manner [80].

**Figure 3:**
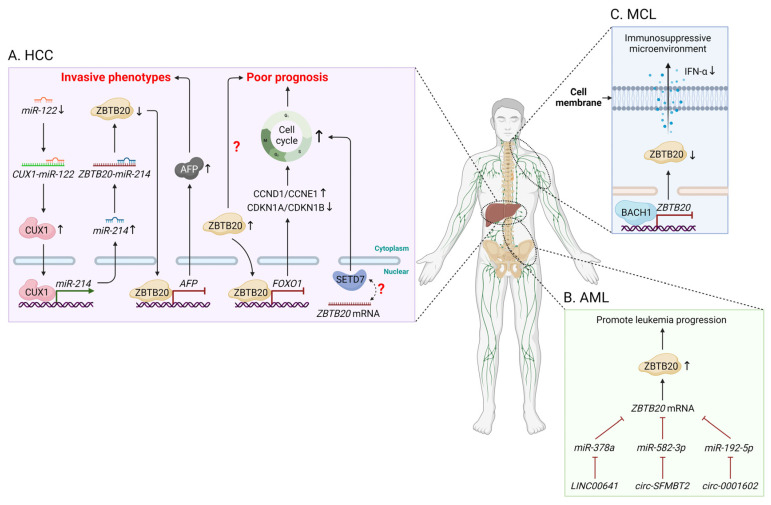
The function of ZBTB20 in human HCC and hematological malignancies. (**A**) Decreased miR-122 expression in HCC leads to an increase in CUX1 protein expression, resulting in repression of ZBTB20 through an increase in miR-214 expression. Repression of ZBTB20 further leads to elevated AFP expression and aggressive tumor behavior. Conversely, increased ZBTB20 expression has been revealed as an independent marker for poor prognosis in patients with HCC. One mechanism is that ZBTB20 promotes cell cycle progression via suppressing *FOXO1*, which in turn leads to the upregulation of CCND1 and CCNE1 as well as the downregulation of CDKN1A and CDKN1B. On the other hand, ZBTB20 promotes cell cycle progression via correlation with SETD7, but it remains unclear how they interact with each other. (**B**) ZBTB20 promotes AML progression via multiple pathways, including the LINC00641/miR-378a/ZBTB20 axis, circ-SFMBT2/miR-582-3p/ZBTB20 axis, or circ-0001602/miR-192-5p/ZBTB20 axis. (**C**) In MCL cells, *ZBTB20* is a novel downstream target repressed by BACH1, which suppresses ZBTB20-driven IFN-α production, creating an immunosuppressive tumor microenvironment. AFP, alpha-fetoprotein; AML, acute myeloid leukemia; BACH1, BTB and CNC homology 1; CCND1, cyclin D1; CCNE1, cyclin E1; CDKN1A, cyclin-dependent kinase inhibitor 1A; CDKN1B, cyclin-dependent kinase inhibitor 1B; circ-SFMBT2, circular RNA Scm-like with four mbt domains 2; CUX1, Cut homeobox 1; *FOXO1*, forkhead box O1; HCC, hepatocellular carcinoma; IFN-α, interferon α; MCL, mantle cell lymphoma; miR, microRNA; SETD7, SET domain-containing 7.

In contrast to mice, ZBTB20 has also been implicated in the reactivation of AFP in human HCC cells via a microRNA (miR)-122-mediated regulatory axis, in which ZBTB20 acts as a negative regulator of invasive phenotypes [83] (Figure 3A). Conversely, other studies revealed the increased ZBTB20 expression as an independent marker for poor prognosis in patients with HCC [84,85]; however, it remains unclear how increased ZBTB20 expression affects prognosis. One mechanism is that ZBTB20 promotes cell cycle progression via suppressing forkhead box O1 (*FOXO1*), which in turn leads to the upregulation of cyclin D1 and cyclin E1 as well as the downregulation of cyclin-dependent kinase inhibitor 1A (CDKN1A) and CDKN1B [85]. ZBTB20 also participates in human HCC development and progression via correlation with SET domain-containing 7 (SETD7), a histone lysine methyltransferase involved in inflammation, metabolism, and oncogenesis [86]. On the other hand, ZBTB20-mediated regulation represents an important mechanism responsible for infection-associated HCC. For example, depletion of ZBTB20 in Huh7 human hepatocytes increases the percentage of hepatitis C virus (HCV)-infected cells and HCV production, indicating that ZBTB20 suppresses HCV infection [75], whereas in HCC patients with chronic infection of hepatitis B virus (HBV), HBV DNA integrates into *ZBTB20*, which is upregulated in tumor tissues and associated with HBV integration frequency [87]. These findings imply distinct ZBTB20-mediated regulation in human HCC caused by different hepatitis viruses, a topic that requires further exploration.

### 4.2. Gastric Cancer

Based on a genome-wide association study from 3,279 individuals of Chinese descent, *ZBTB20* rs9841504 (intron variant, C > G/T) on 3q13.31 was quickly identified as a new susceptibility locus for non-cardia gastric cancer (GC) [88]. Further analysis from 1273 subjects in a Chinese population revealed that rs9841504 is associated with severe intestinal metaplasia/dysplasia, suggesting a potential role of ZBTB20 at an early stage of gastric carcinogenesis [89]. However, no association was found between rs9841504 and GC risk in both Korean and Hispanic patients [90,91], reflecting an ethnic-specific distribution of *ZBTB20* variants. Instead, a novel genetic variant of rs758277701 (missense variant, G > A) was found in the microsatellite instability (MSI) subtype of GC in a Korean population [92], and another variant of rs9288999 (intron variant, G > A) was further identified as a protective factor for reducing GC risk in the Chinese Han population [93]. Despite efforts to identify *ZBTB20* variants in GC, little is known about their roles during the progression of GC, and the same is true for ZBTB20 WT protein. To date, only one study has explored the effect of ZBTB20 on the regulation of human GC cells. It showed that silencing ZBTB20 in GC cell lines not only inhibits cell proliferation but also represses cell invasion and migration, while overexpression of ZBTB20 exhibits the opposite patterns, suggesting an oncogenic role of ZBTB20 in GC; importantly, the ZBTB20-induced phenotypes in GC cells is mainly mediated by NFKBIA/NF-κB signaling pathway [94].

### 4.3. CNS Cancer

Like GC, analyses from human glioma samples have identified the *ZBTB20* gene as a hot mutation site [95,96]. MicroRNAs (miRNAs) that target *ZBTB20* and the long non-coding RNA (IncRNA) *ZBTB20-AS4* are also implicated in the classification and prognosis of patients with low-grade gliomas [97,98,99]. In patients with Sonic Hedgehog medulloblastoma (Shh-MB), fusion transcripts of *ZBTB20* have been identified as recurrent fusions in Shh-MB [100]. These findings, together with the physiological function of ZBTB20 in neurodevelopment, suggest that ZBTB20 is likely to be a key regulator of CNS cancers. Mechanistically, ZBTB20 promotes glioblastoma progression via the miR-758-5p/ZBTB20 axis or by itself [101], but the mechanism by which ZBTB20 exerts roles on glioblastoma progression remains unknown.

### 4.4. Blood Cancer

Since the early discoveries of ZBTB16 in APL and BCL6 in B-cell lymphomas and leukemias, an increasing number of studies have focused on the roles of ZBTB proteins in hematological malignancies. In a very early study, microarray analysis from patients with B-cell chronic lymphoblastic leukemia (B-CLL) identified *ZBTB20* as the top differentially expressed gene in terms of VH mutation status, but its function in B-CLL was not specified [102]. By contrast, *ZBTB20* was verified as a direct target of miR-378a in acute myeloid leukemia (AML) cells, and the miR-378a/ZBTB20 is further under the modulation of a novel AML-related IncRNA LINC00641 to promote cell growth and migration [103]. Later, another study discovered that *ZBTB20* mRNA levels are dramatically elevated in AML patients and cell lines; silencing ZBTB20 significantly suppresses malignant behaviors of AML cells, which is regulated by a circRNA/miRNA-mediated axis [104]. Likewise, ZBTB20 was further identified to exert a tumor-promoting role in AML cells via the circ-0001602/miR-192-5p/ZBTB20 axis [105] (Figure 3B). Despite these findings, none of the studies delineate the in vivo effects of ZBTB20 on leukemia progression, nor is it clear whether ZBTB20 is an original cause or just a mediator of other causative factors in leukemias.

Very recently, one group for the first time revealed the roles of ZBTB20 in mantle cell lymphoma (MCL), an aggressive subtype of non-Hodgkin’s lymphomas [106]. In this study, *ZBTB20* was identified as a novel downstream target repressed by BTB and CNC homology 1 (BACH1), a crucial transcriptional repressor that regulates multiple cellular processes including intracellular heme homeostasis and immune response. Of interest, ZBTB20 triggers increased production of IFN-α in MCL cells upon BACH1 silencing, indicating that ZBTB20 is very likely to participate in the BACH1-mediated regulation of the tumor immune microenvironment (Figure 3C). Although the precise role of ZBTB20 in MCL cells as well as the mechanisms underlying ZBTB20-induced IFN-α production were not investigated in detail, it highlights the importance of ZBTB20 in MCL progression. On the other side, since IFN-α has been applied to MCL treatment [107,108], targeting the BACH1/ZBTB20/IFN-α regulatory axis may provide a novel therapeutic strategy against MCL.

### 4.5. Other Cancers

In patients with primary estrogen receptor α-positive (ERα^+^) breast cancer (BC), *ZBTB20* has been identified as a significantly downregulated gene in biopsies upon treatment of aromatase inhibitors [109], whereas in ERα^+^ BC cell lines, *ZBTB20* is upregulated after treatment of anacardic acid, emphasizing that the roles of ZBTB20 could be treatment- and context-specific in BC [110]. In terms of mechanism, ZBTB20 was only implicated in two non-coding RNA-mediated axes in BC cells [111,112]. Apart from BC, *ZBTB20* rs10511330 (intron variant, T > A/C) and rs16822593 (intron variant, G > A) were identified as two of the top ten single nucleotide polymorphisms (SNPs) in a cohort of patients with colorectal cancer [113]. Multi-genomic analysis from the Cancer Genome Atlas (TCGA) also identified *ZBTB20* as one of ten potential driver genes in cervical cancer [114]. In non-small cell lung cancer (NSCLC), ZBTB20 is upregulated in NSCLC tissues, and it promotes cancer cell proliferation by repressing *FOXO1* [115]. ZBTB20 is also increased in cells that migrate into omentum tissue pretreated with extracellular vesicles isolated from ascitic supernatant of high-grade ovarian cancer patients [116]. Despite the above implications in many types of human cancers, ZBTB20 and its regulatory network remain poorly understood. 

**Table 2:** Overview of ZBTB20 roles in cancers.

Cancer Type			Potential Function/Mechanism	Ref.
HCC	Mice		Transcriptional repression of the *Afp* gene	[78,79]
			(1) Promotion of liver regeneration (2) Regulation of the hepatic expression of Egfr	[80,117]
			One of nineteen highly significant candidate locus implicated in mouse HCC	[82]
	Humans		Reactivation of AFP via the miR-122-mediated regulation	[83]
			An independent marker for poor prognosis in human HCC	[84,85]
			Promotion of HCC by suppressing *FOXO1*	[85]
			Promotion of HCC by correlation with SETD7	[86]
			Suppression of HCV infection	[75]
			Association with HBV integration frequency	[87]
GC	Humans		Identification of rs9841504 as a new susceptibility locus for non-cardia GC in the Chinese population	[88]
			Association of rs9841504 with severe intestinal metaplasia/atypical hyperplasia in the Chinese population	[89]
			Identification of rs758277701 in the MSI subtype of GC in the Korean population	[92]
			Identification of rs9288999 as a protective factor for reducing GC risk in the Chinese Han population	[93]
			Promotion of GC via the NFKBIA/NF-κB signaling pathway	[94]
CNS cancer	Humans	Glioma	A mutation hotspot	[95,96]
		Neuronal and mixed neuronal-glial tumors	Implication of miRNAs that target *ZBTB20* in the classification of pediatric cases	[97]
		Low-grade glioma	Identification of *ZBTB20-AS4* as a critical IncRNA for predicting the prognosis	[98,99]
		Shh-MB	Identification of the fusion transcripts of *ZBTB20* as recurrent fusions	[100]
		Glioblastoma	Promotion of glioblastoma through the miR-758-5p/ZBTB20 axis or by ZBTB20	[101]
Blood cancer	Humans	B-CLL	The top differentially expressed gene in terms of VH mutation status	[102]
		AML	Promotion of cell growth and migration via the LINC00641/miR-378a/ZBTB20 axis	[103]
			Promotion of malignant phenotypes via the circ-SFMBT2/miR-582-3p/ZBTB20 axis	[104]
			Promotion of leukemia development via the circ-0001602/miR-192-5p/ZBTB20 axis	[105]
		MCL	(1) A novel downstream target repressed by BACH1 (2) Involvement in the BACH1-mediated regulation of tumor immune microenvironment	[106]
Others	Humans	BC	Downregulation in ERα^+^ BC biopsies upon treatment of aromatase inhibitors	[109]
			Upregulation in ERα^+^ BC cell lines upon anacardic acid treatment	[110]
			Promotion of cell migration and invasion via the SNHG8/miR-634/ZBTB20 axis	[111]
			Promotion of cell proliferation, migration, and invasion via the circ-0104345/miR-876-3p/ZBTB20 axis	[112]
		Colorectal cancer	Identification of rs10511330 and rs16822593 as two of the top 10 SNPs in patients	[113]
		Cervical cancer	One of ten potential driver genes	[114]
		NSCLC	(1) Upregulation in NSCLC tissues (2) Promotion of cell proliferation by repressing *FOXO1*	[115]
		Ovarian cancer	Increase in cells that migrate in omentum tissue pretreated with extracellular vesicles isolated from ascitic supernatant of high-grade patients	[116]

Note: AML, acute myeloid leukemia; AFP/Afp, alpha-fetoprotein; BACH1, BTB and CNC homology 1; BC, breast cancer; B-CLL, B-cell chronic lymphoblastic leukemia; circ-SFMBT2, circular RNA Scm-like with four mbt domains 2; Egfr, epithelial growth factor receptor; ERα^+^, estrogen receptor α positive; *FOXO1*, forkhead box O1; GC, gastric cancer; HBV, hepatitis B virus; HCC, hepatocellular carcinoma; HCV, hepatitis C virus; IncRNA, long non-coding RNA; miRNA, microRNA; MCL, mantle cell lymphoma; MSI, microsatellite instability; NSCLC, non-small cell lung cancer; Ref., references; SETD7, SET domain-containing 7; Shh-MB, Sonic Hedgehog medulloblastoma; SNHG8, small nucleolus RNA host gene 8; SNPs, single nucleotide polymorphisms.

## 5. Summary and Prospects

To date, there have been many unresolved issues related to the dysregulation of gene transcription and the mechanisms underpinning complex regulatory network in human cancers; ZBTB20 is one of them. Genetically, *ZBTB20* is a hotspot of genetic variation or fusion in many cancers including GC, glioma, MB, colorectal cancer, and cervical cancer. We also found several recurrently occurring mutations in *ZBTB20* in many other cancers when we checked the Cosmic database using the ProteinPaint website (https://proteinpaint.stjude.org/, accessed on 29 January 2024), such as C120Y in ampullary carcinomas and T690fs in gastrointestinal cancers, metastatic prostate cancer, etc. Although the existing studies have barely explored the roles of these variants, we speculate that they are very likely to cause a toxic gain-of-function in human cancers due to the fact that heterozygous *ZBTB20* variants in Primrose syndrome possibly lead to a dominant-negative effect rather than haploinsufficiency [68]. On the other hand, ZBTB20, as well as being a TF, is itself concurrently modulated by different types of non-coding RNAs (e.g., lncRNA, circRNA, and miRNA) and other TFs, highlighting the complexity of ZBTB20-involving regulation in cancer cells. In this context, it will be of tremendous interest to explore the upstream and downstream regulatory network of ZBTB20 in cancer cells. Of note, more attention should be paid to the epigenetic modifications of the *ZBTB20* gene in cancer cells since the gene expression of *ZBTB20* is differentially downregulated in HCC cells after knockdown of histone lysine methyltransferase SETD7 [86]. Last but not least, considering the close link between ZBTB20 and immune regulation in scenarios of both health and cancer, ZBTB20 is possibly involved in the regulation of host immune surveillance and tumor microenvironment. As such, further efforts are needed to delineate the immune responses elicited by ZBTB20 in cancer cells.

Collectively, ZBTB20 plays fundamental roles in the regulation of cellular development and differentiation, metabolism, and immune response. Regardless of the recent advances in understanding of ZBTB20 in cancers, there are still many unaddressed issues. For example, what is the function of *ZBTB20* variants in cancer cells? Is it a loss- or a gain-of-function? In terms of ZBTB20 WT protein, how is it transcriptionally or epigenetically regulated in cancer cells? What is the landscape of ZBTB20-mediated regulation? How do these alterations affect tumor initiation and progression? To answer these questions, further in-depth characterization of ZBTB20 in clinical samples, along with in vivo cell/tissue-specific manipulation of *ZBTB20*, will likely provide an insight into tumorigenesis and cancer progression, thus offering evidence-based guidelines for targeted therapy.

## Figures and Tables

**Table 1:** Overview of the physiological roles of ZBTB20.

Physiological Role		Tissue/Cell Type	Potential Mechanism	Ref.
Lymphoid development and differentiation	Mice	B1, GC B, mature BMPC	Promotion of plasma cell differentiation	[7]
		B cell and plasma cell	Regulation of long-term antibody production and plasma cell persistence after alum-adjuvanted immunization	[8]
		CD8^+^ T cell	(1) Regulation of mitochondrial metabolism and glycolysis (2) Restriction of memory CD8^+^ T cell differentiation (3) Restriction of anti-tumor immunity	[9]
			Transcriptional and epigenetic regulation of memory CD8^+^ T cell differentiation	[64]
		Breg cell	A potential marker gene for Breg cells	[65]
		Intestinal Treg cell	Modulation of intestinal homeostasis	[66]
	Humans	BM, LN, thymus, PBC, fetal liver	A certain role in hematopoiesis, immune responses, and oncogenesis due to its high homology to *BCL6*	[2]
Cellular metabolism	Mice	Liver	Regulation of transcriptional profiling of genes in glucose metabolism	[3]
		Pancreatic β cell	Regulation of glucose sensing and insulin secretion by repressing *Fbp1*	[5]
		Hepatocytes	Regulation of plasma triglyceride metabolism by repressing transcription of lipoprotein lipase	[67]
	Humans	Multisystem	Regulation of lipid and glucose metabolism as well as mitochondrial function	[68,69]
Neurodevelopment	Mice	Hippocampal neuron, cerebellar granule neuron, macroglia	Characterization of two isoforms, *Zbtb20(S)* and *Zbtb20(L)*, in CNS	[63]
		Immature cortical neuron	Regulation of Hi-like cortical neurogenesis	[70]
		Whole brain	Modulation of the sequential neurogenesis in developing cortex	[71]
		Hippocampal neuron	Specification of CA1 field identity in the developing hippocampus	[62]
			Regulation of Hi-dependent long-term memory in mature CA1 neurons	[72]
		Anterior pituitary	Regulation of anterior pituitary development and lactotrope specification	[73]
			Regulation of prolactin expression and lactotrope function	[74]
Immune response and inflammation	Mice	Macrophage	Full activation of TLR signaling and TLR-triggered innate immune response by suppressing the *Nfkbia* gene transcription	[6]
		Spleen cell	Regulation of T cells involved in acute heart allograft rejection through the activation of NF-κB pathway	[76]
		Lymphocyte	Identification of *circZbtb20* as a regulator of ILC3 homeostasis and function	[77]
	Humans	528 lymphoblastoid cell lines	Regulation of multiple infection-related phenotypes	[75]

Note: BM, bone marrow; BMPC, bone marrow plasma cell; Breg, regulatory B; CA1, Cornu Ammonis 1; circ, circular; CNS, central nervous system; *Fbp1*, fructose-1,6-bisphosphatas; GC, germinal center; Hi, hippocampus; ILC3, type 3 innate lymphoid cell; LN, lymph node; PBC, peripheral blood cell; Ref., references; TLR, Toll-like receptor; Treg, regulatory T.
